# Absence of Dr. Billings

**Published:** 1885-01

**Authors:** 


Absence of Dr. Billings.—Through an oversight we failed to announce in our last issue the departure for Europe, in September, of our associate, Dr. Billings.

The doctor is now in Berlin pursuing the object of his journey, which is to make a thorough study of all that pertains to the germ theory of disease, especially the best methods of cultivating bacteria. In this he receives the direct aid of Professors Virchow and Koch.

In taking this step Dr. Billings seems to have foreseen the importance of the knowledge which he has gone to seek. In a paper on the probable epidemic of Asiatic cholera in this city, Dr. E. C. Wendt calls attention to the necessity of efficient action by the health authorities, and says: "In view of recent discoveries it now seems possible to decide positively, and in a very short time, whether we are dealing with the benign or the malignant disease."

He advises the government to send a number of medical men to Berlin to learn the latest methods of bacteria investigation, with a view of introducing bacterioscopy as a special study in the medical schools of this country.

Moreover, we all know how much skepticism exists even among scientific men on this subject. The other day one of these latter, to show the absurdity of Koch's doctrine, swallowed a couple of million of *comma-bacilli* without inducing the slightest choleraic symptoms. Did this prove the benignancy of the microbe? Not at all. It simply showed that one of the necessary factors in the production of the disease was wanting, viz: a proper soil. The man was in good hygienic condition, or he may have taken *comma-bacteria* instead of *comma-bacilli*, the former, according to Koch, being found in simple cholera morbus, while the latter are peculiar to the Asiatic type. Here we see the value of microscopical investigations. We also arrive at the conclusion that since on the nature of the soil, as well as on the malignancy of the microbe depends the production of the disease, strict sanitary measures should be enforced in order to make that soil germ-proof, and quarantine regulations established which will prove barriers to the entrance of the terrible *bacillus*.

Desiring for the doctor complete success in his undertaking, we look forward with pleasure to his return and with interest to the publication in this JOURNAL of the results of his labors.

